# Adding Years to Your Life (or at Least Looking Like It): A Simple Normalization Underlies Adaptation to Facial Age

**DOI:** 10.1371/journal.pone.0116105

**Published:** 2014-12-26

**Authors:** Sean F. O'Neil, Amy Mac, Gillian Rhodes, Michael A. Webster

**Affiliations:** 1 Department of Psychology, University of Nevada, Reno, Nevada, United States of America; 2 ARC Centre of Excellence in Cognition and its Disorders, School of Psychology, University of Western Australia, Perth, Australia; Monash University, Australia

## Abstract

Adaptation has been widely used to probe how experience shapes the visual encoding of faces, but the pattern of perceptual changes produced by adaptation and the neural mechanisms these imply remain poorly characterized. We explored how adaptation alters the perceived age of faces, a fundamental facial attribute which can uniquely and reliably be scaled by observers. This allowed us to measure how adaptation to one age level affected the full continuum of perceived ages. Participants guessed the ages of faces ranging from 18–89, before or after adapting to a different set of faces composed of younger, older, or middle-aged adults. Adapting to young or old faces induced opposite linear shifts in perceived age that were independent of the model's age. Specifically, after adapting to younger or older faces, faces of all ages appeared 2 to 3 years older or younger, respectively. In contrast, middle-aged adaptors induced no aftereffects. This pattern suggests that adaptation leads to a simple and uniform renormalization of age perception, and is consistent with a norm-based neural code for the mechanisms mediating the perception of facial age.

## Introduction

Adaptation aftereffects have played a prominent role in studies of face perception [1], [2]. After viewing a distorted face, a normal face looks distorted in the opposite way [3]. Robust aftereffects occur for perceptual dimensions along which faces naturally vary, thus affecting many of the cognitive and social judgments made about faces. These include biases in perceived identity, gender, ethnicity, or expression, as well as in attributes such as attractiveness or trustworthiness [4]–[7].

**Figure 1:**
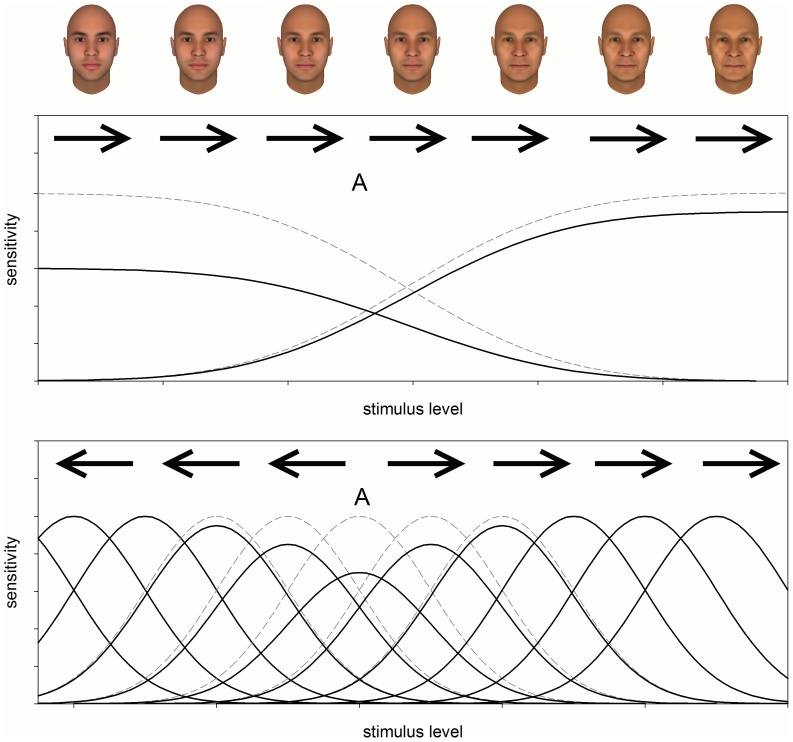
Models of face coding and aftereffects. Norm-based (top) vs. exemplar-based (bottom) models of face coding and aftereffects. These differ in whether the levels of the face (e.g. age) are encoded by differences in two broadly tuned mechanisms or by the peak response within narrowly-tuned channels. Channel sensitivities are depicted before (dashed), or after (solid) adapting to stimulus level A. Arrows show the predicted direction of aftereffects that adaptation to A induces in different ages.

**Figure 2:**
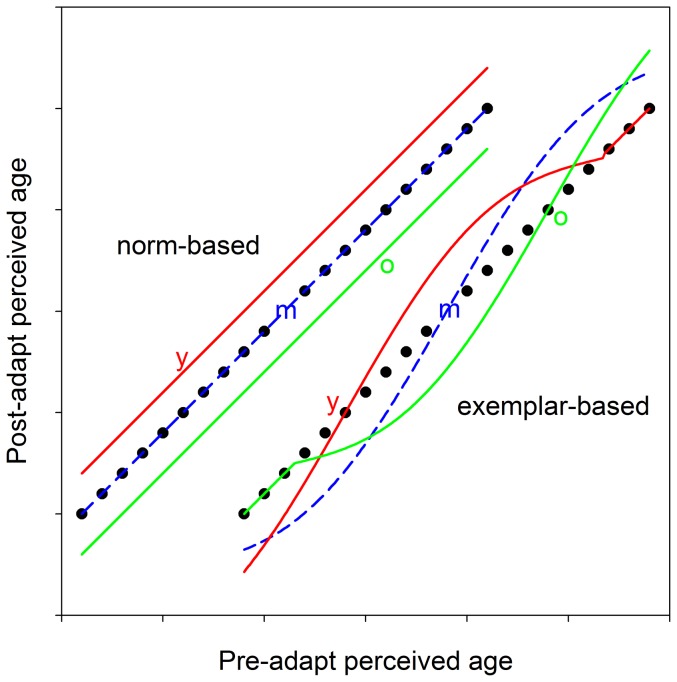
Age aftereffects predicted by each model. Aftereffects predicted by norm-based (left) vs. exemplar-based (right) representations. Each plot illustrates judgments of perceived age before adaptation (solid black circles) or after adapting to young (y: red solid lines) or old (o: green solid lines) faces or to middle-aged faces (m: blue dashed lines) that are near the observer's old/young categorical boundary. The norm-based model shifts all ages to appear older (y adapt) or younger (o adapt) while predicting no change for adaptation to the norm (m). The exemplar model instead predicts no shifts at any adapting level while older and younger faces appear biased away from the adapt level. Note the range of ages affected depends on how narrowly or broadly tuned the channels are.

Numerous studies have focused on the specific form of these aftereffects to infer the coding scheme underlying face perception. Two prominent alternatives are relative (norm-based) codes, in which individuals are represented by how they deviate from a prototype; and absolute (exemplar) codes, in which faces are encoded by template matching (Fig. 1) [8]. These differ in whether the average face has a special status, and predict different patterns of adaptation [1], [2] (Fig. 2). In norm-based codes, adaptation to an individual face renormalizes percepts so that the adapting face appears less distinctive. This tends to recenter “face space” nearer to the adapting level and induces shifts in the appearance of all faces in the same direction. It also predicts no aftereffects when adapting to the average face (since it is already the norm). In exemplar codes, adaptation instead leads to more localized aftereffects by reducing sensitivity in channels tuned to the adapt face. This predicts no perceived shift in the adaptor itself, while other faces look less like the adaptor. Thus opposite aftereffects occur for faces on opposite sides of the adaptor. This model also differs in that the average face is coded by a template in the same way as other faces and thus adaptation to the average should lead to similar aftereffects as adaptation to other ages.

Several previous studies have pointed to norm-based coding from the patterns of face aftereffects. For example, adapting to distorted faces biases the appearance of undistorted faces but not vice versa, an asymmetry consistent with coding the distortions relative to the (undistorted) norm [3]. Moreover, when adapting to distorted faces such as an expanded face, all faces – including the adapting face – appear less expanded, consistent with a renormalization of the adapting distortion [6], [9], [10]. Face identity aftereffects are stronger between pairs of faces that lie on opposite sides of the average, suggesting that faces and their “antifaces” are coded as opposite directions relative to a norm [11]; and aftereffects are also larger for faces that are more distinctive, presumably because these are farther from the norm [12], [13].

However, whether norm-based coding underlies most facial attributes remains uncertain. Head orientation (view) and gaze direction show aftereffects consistent with exemplar coding with three or more channels, with one tuned to the direct view [14], [15]. Moreover, recent studies have argued against renormalization for gender because the adapting gender itself may not become less distinctly male or female as observers adapt to it [10], and because the aftereffects may not increase monotonically with increasing physical deviations of faces from androgyny [16] (though the falloff in aftereffects may only occur outside the range over which face gender naturally varies [17]). Finally, recent models have questioned whether adaptation can in principle distinguish between norm-based and exemplar codes [18]. Thus both the form and implications of face aftereffects for neural coding remain actively debated.

One factor hindering understanding of face adaptation is that aftereffects are usually only assessed for a limited range of faces, often near the prototype or a category boundary (e.g. male vs. female). This is because it is easier for participants to say *whether* a face is male or female, than *how* male or female it appears. However, from these restricted measures it is difficult to evaluate how adaptation is altering percepts of the facial dimension. Rating scales have been used to more fully explore the response changes, but have been limited to coarse sampling of unnatural dimensions such as distortions [6].

We took advantage of one attribute where fine metric judgments about the face can be made – facial age. Age is one of the most salient and ecologically important characteristics of the face, and guessing someone's age is not only routine and intuitive but can be done with good accuracy for faces throughout the lifespan [19], [20]. Thus age estimates provide a unique and sensitive psychometric scale for probing a fundamental dimension of face perception over the entire range of natural variation. Previous studies have shown that perceived age can be strongly biased by adaptation and that the aftereffects are driven by both the textural and shape changes that accompany aging [21]–[23]. They have also suggested that adaptation to age partly reflects higher levels of visual coding [24]. However, this work has again been limited to assessing how adaptation to young or old faces alters the appearance of intermediate ages. Here we probed the effects of adapting to faces of three different ages (young, middle-aged and old) on the appearance of ages across the entire span of adult ages. This allowed us to assess specifically how adaptation alters the visual code for age, and thus to determine whether the code is more consistent with a norm-based or exemplar-based representation.

## Methods

### Participants

Observers were students (aged 20–30) at the University of Nevada. One group (n = 19) adapted to young and old faces in counterbalanced sessions. A separate group (n = 9) adapted to middle-aged faces. Written consent was obtained from observers under protocols approved by the University of Nevada IRB.

### Stimuli

Stimuli were digital color photographs of neutral-expression, Caucasian faces with recorded ages from a public database [25]. To reduce age cues from hair [26], male images were clean shaven and all faces were cropped with an oval window (3∶4 ratio). Eighty images served as test faces and 30 as adapting faces. The test faces were relatively evenly spaced from 18 to 89 years with similar numbers of males (38) and females (42). Adapt faces were a different set of 10 younger (M = 34.3, SD = 2.54) or older (M = 65.0, SD = 1.33) individuals, with 5 from each gender. A third group of 10 middle-aged faces was also used, based on the participants' ratings (see below).

### Procedure

Stimuli were presented on a CRT monitor in a darkened room. During testing, the 80 test faces were displayed in random order for 500 ms, and observers had 4 sec after each to enter the estimated age using a handheld keypad. Participants were told only that ages fell within the 2 digit range of 10–99 years. A practice trial with different faces was provided to familiarize them with the task. To measure the effects of adaptation observers first completed a session where they judged the ages while adapted to a uniform gray screen (pre-adapt), and then a separate session where the same faces were judged while adapted to one age group (post-adapt). During face adaptation, the 10 adapt faces were shown in a random sequence of 500 ms each for an initial period of 2 minutes, and a new random sequence was repeated during the 5 sec between each test. To limit low-level aftereffects, adapt faces (subtending ∼6.4°×8.3°) were shown 1.5 times larger than test faces (∼4.3°×5.5°) and were jittered in position. The jitter arose from the fact that the photos were cropped with a uniform template centered on the face of each individual. Because the individual faces differed in how well centered they were on the screen, the location of the presented faces (but not the invisible frame) varied randomly and typically over a small fraction of the face widths.

The group of observers tested with the middle-age adapt set completed a prior additional session to estimate their age boundary for classifying an adult face as old or young. The 80 test faces were shown in random order, and categorical responses were made to indicate whether each appeared “young” or “old.” A probit function was fit to the responses to determine the old/young category boundary. This averaged 47 yrs (SD = 6.7), and defined the middle adapting age.

## Results

**Figure 3:**
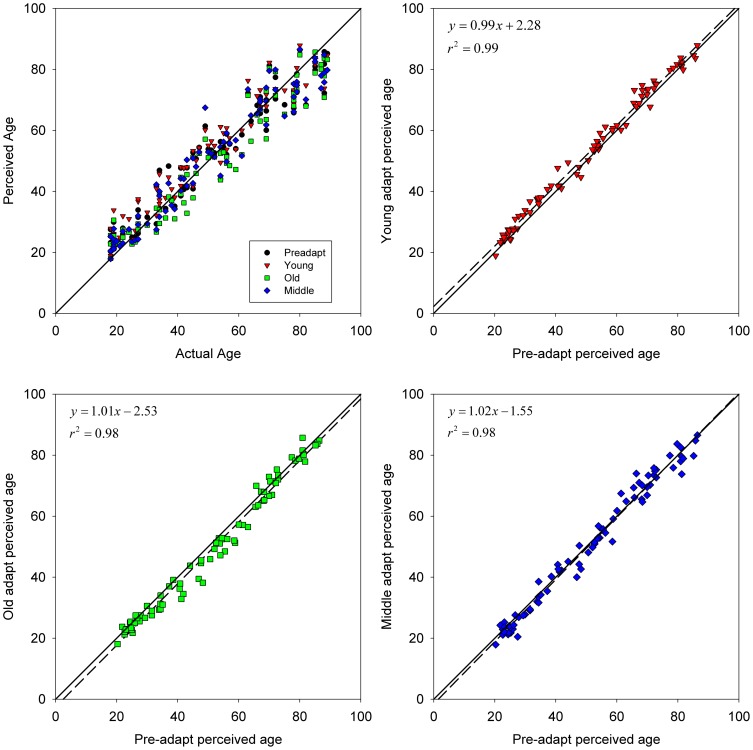
Results of adapting to age. (a) Perceived age vs. physical age before (pre-adapt: black circles) or after adapting to young (red triangles), middle (blue diamonds) or old (green squares) ages. (b) Perceived age after vs. before adapting to young faces (symbols). Lines show the linear regression (dashed) and unit diagonal (solid); intercept = 2.15, t(78) = 3.25, p = .002; slope = 0.99, t(78) = 0.65, NS. (c) Old adapt; intercept = −2.56, t(78) = −2.97, p = .004; slope = 1.01, t(78) = 0.63, NS. (d) Middle-age adapt; intercept = 0.76, t(78) = 0.815, NS; slope = 0.99, t(78) = 1.80, NS.

Observers were highly reliable at judging age from the photos. Physical age accounted for ∼94% of the variance in the age estimates (Fig. 3a), with an average inter-observer standard deviation of 9 years in the pre-adapt settings. This pattern closely replicates the results of a previous study by Burt and Perrett [19], who similarly found that physical age explained 94% of the variance in judging the age from images of adults ranging from 20 to 60 years. Moreover, in our study much of the residual variance in the average settings was not because observers were inconsistent, but because some models did not look their real age. This factor was removed by plotting post-adapt settings as a function of pre-adapt settings (Fig. 3b–d), which eliminated almost all of the scatter (r^2^>0.98).

Regression lines fitted to the settings reveal a simple and systematic effect of adaptation. Adapting to the young (Fig. 3b) or old (Fig. 3c) faces shifted the intercepts up or down by ∼2.5 years, a small bias relative to the wide physical range tested but nevertheless highly significant. In contrast, adaptation did not introduce a measurable change in the fitted slopes. Thus, the aftereffect is well approximated by a constant bias: after adapting to younger (older) faces, *everyone* looked a few years older (younger). The magnitude of the biases we observed is small compared to the aftereffects reported in previous studies of age adaptation (e.g. [21]–[24]). However, this is likely in part due to the fact that in the present study, the adapt and test faces differed in identity and that the ensemble of adapting faces included both genders. Age aftereffects [21], [23], [24], and face aftereffects in general [1], show partial selectivity for gender and identity, and gender selectivity has also been observed for adaptation to old and young voices [27]. Thus the gender and identity differences in our stimuli would be expected to reduce the transfer of the adaptation. On the other hand, the fact that significant transfer nevertheless occurs is also consistent with these previous studies in suggesting that observers can adapt to the attribute of age independent of the specific individual faces carrying that attribute.

Adapting to the middle-age did not significantly change the slope or intercept (Fig. 3d). The results were thus intermediate to the perceived age biases induced by the young and old adapt ages. This was confirmed by paired t-tests comparing the age settings for each image under the different adaptation conditions. Both young and old adaptors produced biases that significantly differed from the middle age adapt condition (young vs. middle: t(79) = 3.79, p = 0.0001; old vs. middle: t(79) = −5.45, p = 10^−7^) and from the pre-adapt settings (young post vs. pre: t(79) = 7.24, p = 10^−10^; old post vs. pre: t(79) = −6.51, p = 10^−9^) while the settings following middle-age adapt did not differ (middle pre vs. post: t(79) = 1.18, p = 0.24). The aftereffects thus closely followed the predictions for a norm-based code, and reveal a simple linear shift underlying the renormalization. Higher-order terms fit to the post- vs. pre-adapt settings did not (young) or only very marginally (<0.05%, middle and old) increased the explained variance, showing that the aftereffects are ∼fully captured by a linear shift.

## Discussion

We exploited observers' natural expertise in evaluating age to fully characterize how adaptation alters this fundamental dimension of face perception. The aftereffects (after adapting to young or old faces) reflect simple linear shifts in perceived age – literally adding or subtracting a constant to the observers' estimates. Moreover, the adaptation appears to reset these estimates relative to faces that appeared “neutral” in age, and adapting to this neutral age did not significantly alter their judgments. Thus the results are well described as a uniform renormalization of facial age. This simple and uniform recalibration resembles how chromatic adaptation alters color appearance, and reinforces suggestions that the visual system adopts similar norm-based representations for very different stimulus domains [1], [28]. (For color, the sensitivity changes primarily reflect a multiplicative rescaling of the cone photoreceptor signals, which predicts a constant log shift. However, this rescaling results in approximately linear changes in opponent color signals when the cone inputs are opposed, as in the long- vs. medium-wave cone-opponent dimension [29]–[31]. Similarly, the present results cannot discriminate between a multiplicative or additive gain change within the putative channels.)

It remains unknown whether the adaptation pattern for age applies to other facial dimensions. The perception and adaptation of adult age is heavily dependent on texture and thus may differ from other facial judgments that rely more on shape cues [21], [22]. These shape cues may be especially prominent for judging the age of younger faces such as children, for which structural changes are more pronounced than in adults [32]. However, texture plays an important role in face recognition in general, and thus probably represents an important coding dimension for faces [33], [34]. Our results are consistent with similar norm-based code for both textural and configural dimensions.

While we used size differences between adapt and test faces to partly control for low-level interactions, as with most face aftereffects, it remains uncertain to what extent the aftereffects directly reflect explicit face-coding mechanisms [1], [35], [36]. Textural aftereffects can occur for a number of dimensions, from simple color and contrast to attributes such as element density [37]. Analyses of our images showed that mean color or contrast did not covary with age and thus were unlikely to underlie the age judgments. Moreover, these low-level image properties could not in any case be the basis for the aftereffects, since they would not predict opposite aftereffects for old and young adaptors. Opposite color (or texture) aftereffects would require that young and old faces were defined by roughly complementary stimuli (e.g. red vs. green) relative to a neutral stimulus (grey). Instead, the skin tones of all the faces varied in similar directions from gray. Whether other textural dimensions not explicitly associated with face coding might account for the perceived changes in age remains possible. Yet importantly, it is unlikely that these would have a neutral point for the adaptation that was tied to observers' subjective judgments of a neutral middle age. This correspondence between a perceptual norm (the subjective category boundary for old vs. young) and a “neural” norm (the null point for the adaptation), suggests that the perceptual norm does not reflect an arbitrary learned criterion for age, but rather an actual neutral point in how visual sensitivity is calibrated [38].

Regardless of the specific neural sources of our age aftereffects, adaptation clearly affected the coding of stimulus attributes that are important for judging age. Our results show that the adaptation-induced response changes within these mechanisms reflect a simple linear renormalization of the perception of age. More generally, they are consistent with a norm-based neural code for the perception of facial age.

## Supporting Information

S1 File **Observers' perceived age settings before and after adapting to three age ranges.**
(XLSX)
